# *Ptomaphaginus troglodytes* sp. n., the first anophthalmic species of Ptomaphaginina from China (Coleoptera, Leiodidae, Cholevinae, Ptomaphagini)

**DOI:** 10.3897/zookeys.749.24964

**Published:** 2018-04-10

**Authors:** Michel Perreau, Jan Růžička

**Affiliations:** 1 IUT Paris Diderot, Université Paris Diderot, Sorbonne Paris cité, case 7139, 5 rue Thomas Mann, 75205 Paris cedex 13, France; 2 Department of Ecology, Faculty of Environmental Sciences, Czech University of Life Sciences Prague, Kamýcká 129, CZ-165 00 Praha – Suchdol, Czech Republic

**Keywords:** Anophthalmy, China, Guizhou Province, new species, troglobiomorphy

## Abstract

*Ptomaphaginus troglodytes* **sp. n.**, the first anophthalmic species of *Ptomaphaginus* Portevin, 1914 is described from two close caves in Libo Karst, south Guizhou Province, China.

## Introduction

Ptomaphagini is, after Leptodirini, the richest tribe of Cholevinae in species living in subterranean environment (caves or other subterranean habitats). Unlike Leptodirini, in which all species except a few dozen are anophthalmic, cave-dwelling species of Ptomaphagini are at most microphthalmic, a single species is fully anophthalmic. The tribe is presently divided into three subtribes: Baryodirina, Ptomaphagina, and Ptomaphaginina (Perreau 2000). The phylogenetic relevance of this division has been discussed (Gnaspini 1996) but recently confirmed (Antunes Carvalho et al. 2017).

Subterranean Ptomaphagina occur mainly in the Nearctic and Neotropical Regions (Peck 1973, 1984, 1998), but the only fully anophthalmic species, Ptomaphagus (Ptomaphagus) troglodytes Blas & Vives, 1983, occurs in Spain, in the Palaearctic Region (Blas and Vives 1983). All Nearctic cave-dwelling species of Ptomaphagina are at most microphthalmic, even the most troglobiomorphic species *Ptomaphagus parashant* Peck & Wynne, 2013 has remnants of eyes (Peck and Wynne 2013). Microphthalmy in Ptomaphagina has been recently investigated by genetic methods on a population of Ptomaphagus (Adelops) hirtus (Tellkampf, 1844) from the Mammoth cave system in Kentucky, USA (Friedrich et al. 2011; Friedrich 2013). These studies showed the presence of transcripts of all critical components of the phototransduction protein network and a strong photonegative behaviour, which indicate a reduced, but functional visual system.

Ptomaphaginina are mainly distributed in the Oriental Region (Szymczakowski 1964), including the Sunda Islands (Schilthuizen et al. pers. comm.). A single genus with six species, *Proptomaphaginus* Szymczakowski, 1969, lives in Central America (Peck 1983). The Oriental species of Ptomaphaginina belong to three genera: *Ptomaphaginus* Portevin, 1914 (96 species), *Pandania* Szymczakowski (two species) and *Ptomaphaminus* Perreau, 2000 (24 published species + 9 species under description). Some species of *Ptomaphaginus* live preferably in caves (*P. lipsae* Perreau & Lemaire, 2018, *P. otusus* Szymczakowski, 1959, *P. tomellerii* Zoia, 1997) but without significant eye reduction (Szymczakowski 1959, Zoia 1997, Perreau and Lemaire 2018). Most of the species of *Ptomaphaminus* live in caves and many of them have reduced eyes (Perreau 2009; Schilthuizen et al. pers. comm.). Currently, no anophthalmic species of Ptomaphaginina is known, and the purpose of the present paper is to describe the first anophthalmic species of Ptomaphaginina: *Ptomaphaginus troglodytes* sp. n. from Guizhou Province in China.

Guizhou comprises extended karst areas with a high diversity of cave-adapted arthropods and is the Chinese province with the highest number of known troglobitic species (Latella and Hu 2008; Tian and Clark 2012). Most of the known troglobitic Coleoptera from Guizhou belong to highly troglobiomorphic ground beetles, Carabidae: Trechinae (e.g. Deuve 1993, 1995; Deuve et al. 1999; Uéno 2000a, b, 2002; Tian 2009, 2010, 2011, 2013, 2014; Tian and Clarke 2012; Tian et al. 2014a, b, 2017; Tian and Deuve 2016a, b; Huang et al. 2017; Wei et al. 2017; for broader review see Latella and Hu 2008). More recently, three additional papers on troglobiont Staphylinidae: Pselaphinae were published from Guizhou (Yin et al. 2011, 2015; Yin and Li 2015).

## Material and methods

Dissected specimens were relaxed in warm water. Male genitalia were directly dehydrated in ethanol 95% then mounted in Euparal. The female abdomen was cleared in a hot water solution of potassium hydroxide 0.1 N for 10 minutes, then rinsed in distilled water, coloured with Azoblack then dissected to extract the genital segment, which was mounted in DMHF. Photonic microscopic pictures (Figs 15–19) were taken on a Zeiss Axiolab microscope with a Spot Insight IN1820 digital camera. A photograph of the habitus in dorsal view was taken using a Canon macro photo lens MP-E 65mm on a Canon 550D. Multiple layers of focus were combined using Zerene Stacker. High-resolution electronic pictures of external morphology were taken using a Hitachi S-3700N environmental electron microscope at the National Museum, Praha.

Specimens examined are deposited in the following collections:

**JRUC** collection of Jan Růžička, Praha, Czech Republic

**MPEC** collection of Michel Perreau, Paris, France

**NMPC** National Museum, Praha, Czech Republic (M. Fikáček, J. Hájek)

**NSMT** National Museum of Nature and Science, Tokyo, Japan (S. Nomura)

The distribution map was produced and edited in ESRI ArcMap 10.5 of ArcGIS Desktop 10.5 suite. For map layers, free levels 0–2 data from Global Administrative Areas (http://www.gadm.org, ver. 2.8) and Natural Earth (http://naturalearthdata.com, Cross Blended Hypso with Relief, Water, Drains, and Ocean Bottom) were used.

## Taxonomy

### Ptomaphaginus troglodytes sp. n.

Taxon classificationAnimaliaColeopteraLeiodidae

http://zoobank.org/957DADD8-4248-4CCE-874C-68C693144DDA

Figs 1 , 2–5 , 6–11 , 15–19 , 20

#### Type locality.

China: Guizhou Province, Libo Xian County, Shuiboshu Dong cave [ca. 25°29'05"N, 107°52'54"E], 490 m.

#### Material examined.

Holotype male (NSMT): “Shuiboshu Dong cave (490 m) / Shuipu cun [ca. 25°29'05"N, 107°52'54"E], Yuiping Zhen / Libo Xian // (Guizhou, CHINA) / 13.ix.1997, T. Kishmoto [leg.] // HOLOTYPUS / *Ptomaphaginus troglodytes* sp. n. / M. Perreau & J. Růžička, 2018”. Paratypes (NSMT, JRUC, MPEC): 1 male and 2 females, same data; 1 male and 1 female, “Yamen Dong cave [ca. 25°29'N, 107°54'E] / Shuibo Zhai, Shuipu Cun / Libo Xian // (Guizhou, CHINA) / 13.ix.1997, T. Kishmoto [leg.] // PARATYPUS / *Ptomaphaginus troglodytes* sp. n. / M. Perreau & J. Růžička, 2018”.

#### Description.

**Figure 1.**
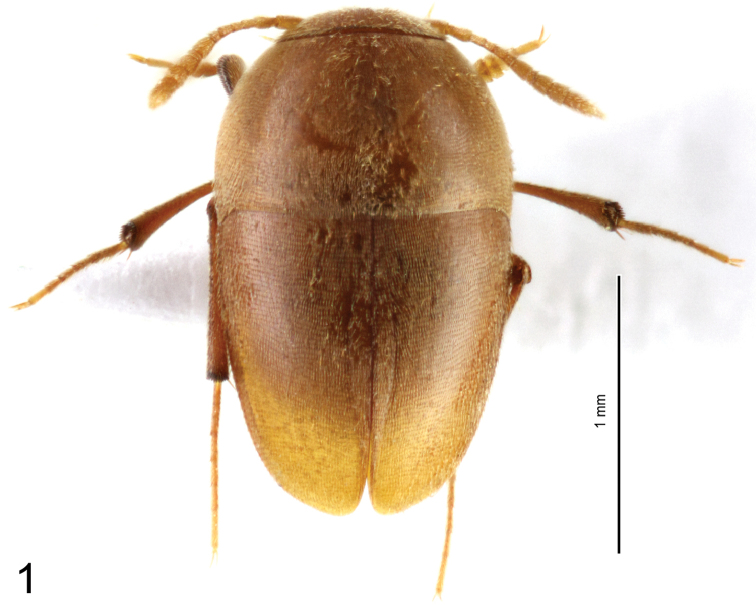
*Ptomaphaginus troglodytes* sp. n., male holotype from Shuiboshu Dong cave, habitus in dorsal view.

**Figures 2–5.**
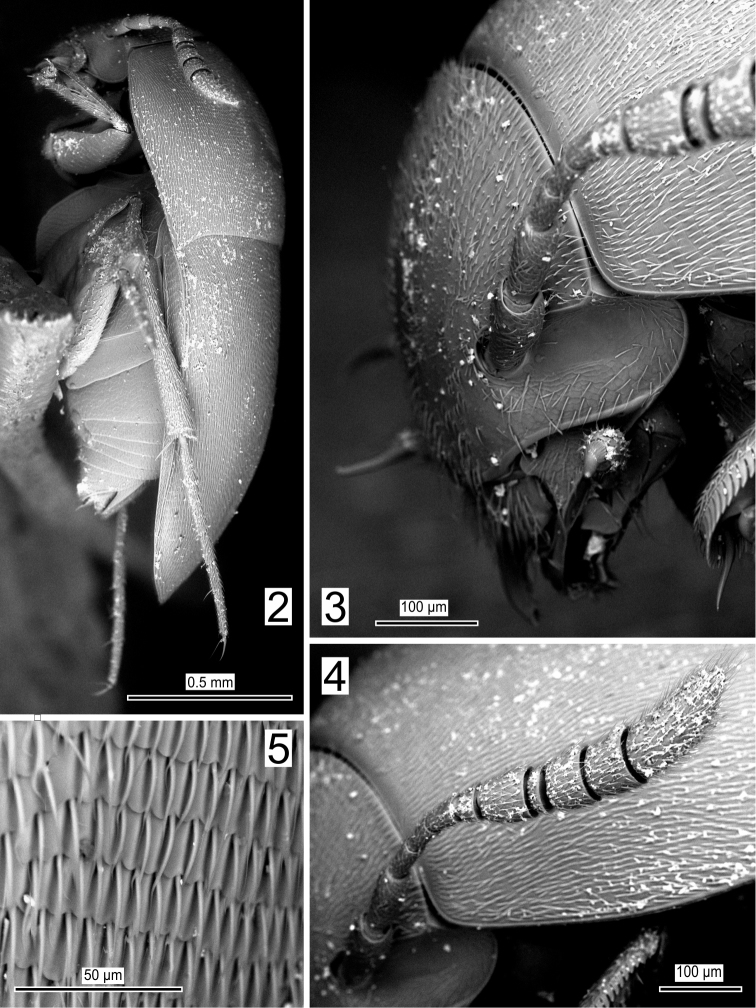
*Ptomaphaginus troglodytes* sp. n., male holotype from Shuiboshu Dong cave, SEM. **2** habitus laterally **3** head laterally **4** left antenna **5** surface of left elytron.

Length 1.85 mm. Body widely ovoid, uniformly light brown (depigmented). Body covered with recumbent setae inserted along tight transverse strigae (Fig. 5). Habitus in Figs 1, 2.

*Head* without eyes (Fig. 3), antenna slender, the club weakly dilated (Fig. 4), the ratio of the lengths of antennomeres to the length of the first one are as follows: 1.00, 0.60, 0.36, 0.26, 0.31, 0.29, 0.45, 0.19, 0.38, 0.45, 1.07. Mandibles with two teeth along the internal side (Fig. 9). Maxillary palpus with the apical segment slender and very elongated, slightly longer than the penultimate (Fig. 8).

*Pronotum* transverse, 1.6 times wider than long, the largest width just before the base. Lateral sides arcuate, the posterior angles clearly protruding behind the posterior margin. Pronotal surface with transverse microstrigae.

*Elytra* short and wide, as long as wide, the greatest width near the base. Surface covered with transverse microstrigae, similar to that of the pronotum (Fig. 5). One incomplete parasutural longitudinal stria, extending over the basal half of the elytral length.

**Figures 6–11.**
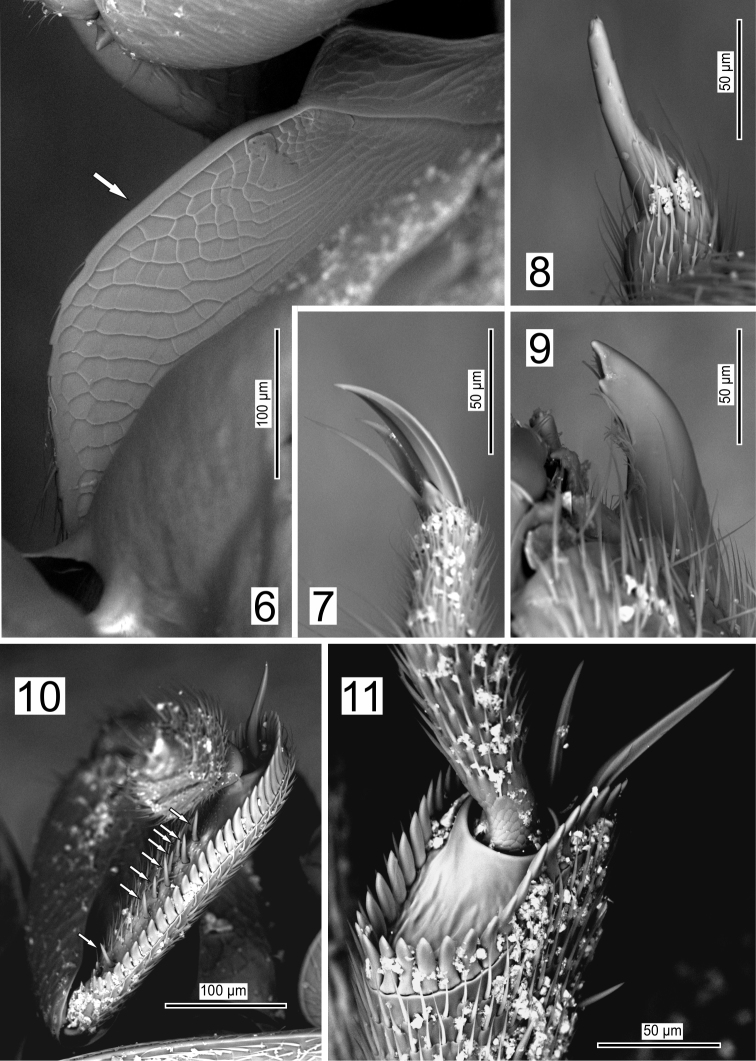
*Ptomaphaginus troglodytes* sp. n., male holotype from Shuiboshu Dong cave, SEM. **6** mesoventral process in lateral view **7** apex of mesotarsus in lateral view **8** apex of maxillary palpus in dorsal view **9** right mandibula in dorsal view **10** left protibia in lateral view (arrows indicate position of spines on ventral side) **11** apex of mesotibia in dorso-posterior view.

*Mesoventral process* with a high, widely rounded medial carina (Fig. 6). Metaventrite with lateral metaventral sutures slightly convergent symmetrically toward the central axis of the body. Metatergum long and thick, extending approximately half the length of the elytra (Fig. 18).

*Protibia* with a row of regular spines along the external side and with a second internal row on the ventral side, with one spine situated basally and a line of seven spines medially (Fig. 10). Mesotibia and metatibia with a comb of equal-sized spines around their apex (Fig. 11). Tarsal formula 5–5–5 in both sexes. Male protarsi widely dilated, as wide as the apex of the protibia. Female protarsi slightly dilated.

*Male genital segment* with a spiculum gastrale extending beyond the anterior margin of epipleurites on one third of its length and slightly narrowed on this part (Fig. 17). Latero-posterior margin of the epipleurites with a row of moderately strong setae (Fig. 17). Aedeagus with parameres fused laterally to the median lobe, tegmen without basal blade, the median lobe 2.6 times as long as wide, the apex roughly triangular in dorsal view (Fig. 15), with two lateral rows of eight strong and long setae inserted perpendicularly to the main plan of the aedeagus, and a preapical lateral row of four tiny setae (Fig. 16). Endophallus with a long, thick stylus, thicker at the base and getting progressively thinner from the base to the apex, with transverse stria in the distal half (Fig. 15).

**Figures 12–14.**
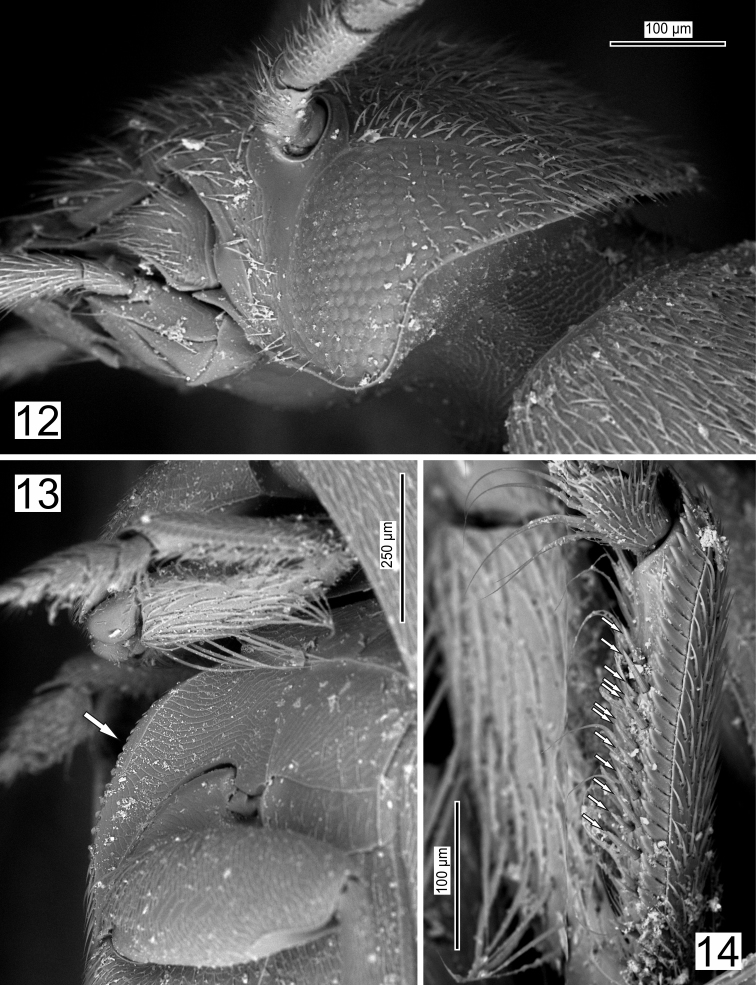
*Ptomaphaginus* sp., male of epigean species (China: Jiangxi province, Jinggang Shan Mts., Baiyinhu env., NMPC). **12** head in lateral view **13** mesoventral process in lateral view **14** left protibia in lateral view (arrows indicate position of spines on ventral side).

**Figures 15–19.**
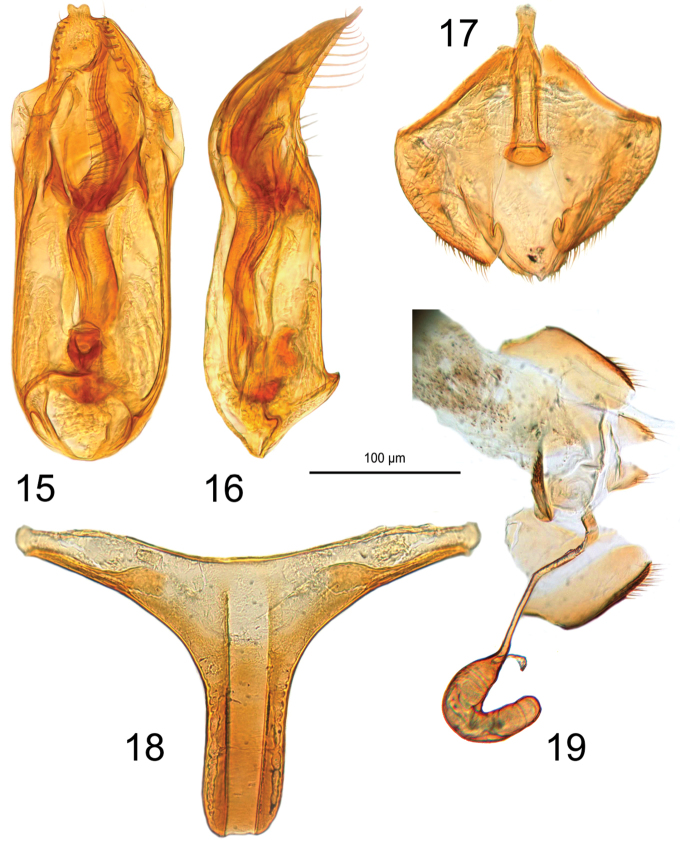
*Ptomaphaginus troglodytes* sp. n., male paratype. **15** aedeagus in dorsal view **16** aedeagus in lateral view **17** male urite IX **18** male metatergum in dorsal view. *Ptomaphaginus troglodytes* sp. n., female paratype **19** female genital segment, with spermatheca.

*Female abdominal segment IX* with short gonocoxites (Fig. 19). Spermatheca uniformly sclerotized, spermiduct short and straight, spermathecal gland small (Fig. 19).

#### Diagnosis.

Distinct from other *Ptomaphaginus* in the absence of eyes, short, wide body shape, especially the elytra as wide as long (taken together), more developed setation of the aedeagus, and transverse microstrigae of the elytra which are extremely tight and orthogonal to the suture, not oblique as generally in Ptomaphagini.

The identification table of Chinese species of *Ptomaphaginus* given in the revision of the genus by Wang and Zhou (2015) should be modified by adding the first couplet before all others:

**Table:** 

1	Anophthalmic. Transverse microstrigae of elytra tight and orthogonal to the suture	***P. troglodytes* sp. n.**
–	Eyes well developed. Transverse microstrigae of elytra more spaced out and oblique	**Other species**

Figs 12–14 illustrate some characters of a Chinese epigean species of *Ptomaphaginus* to compare with *P. troglodytes*: Fully developed eyes (Fig. 12), mesoventral process less elevated (Fig. 13) and ventral face of protibia with the second row of spines more regular (Fig. 14).

#### Etymology.

Cave-dweller in Latin, because of the association of the new species with caves; noun in apposition.

#### Biology and biogeography.

No bionomic details are available for the two small series, collected in Shuiboshu Dong and Yamen Dong caves. This is the first species of *Ptomaphaginus* reported from Guizhou Province, most probably as a result of a gap in knowledge of the fauna of the centre of southern China (see Wang and Zhou 2015: 336, figure 20); this may be improved by additional sampling activities.

#### Distribution.

**Figure 20.**
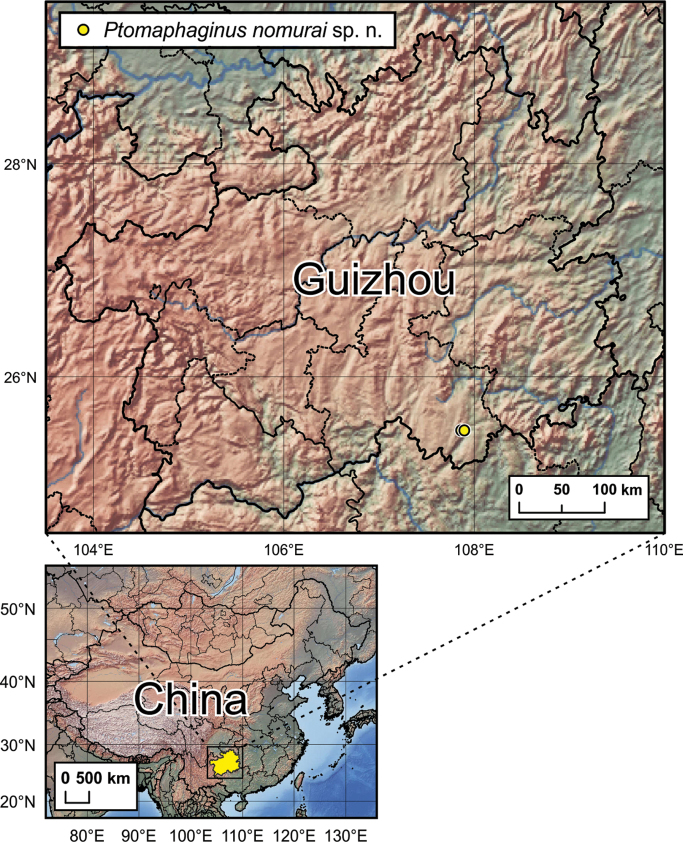
Distribution map of *Ptomaphaginus troglodytes* sp. n. in Guizhou Province, China.

The species is presently known only from two closely situated caves in Libo Karst area, south of Guangxi Province, China (Fig. 20).

## Supplementary Material

XML Treatment for Ptomaphaginus troglodytes
